# Morphology engineering of *Streptomyces coelicolor* M145 by sub-inhibitory concentrations of antibiotics

**DOI:** 10.1038/s41598-017-13493-y

**Published:** 2017-10-16

**Authors:** Hu Wang, Guoping Zhao, Xiaoming Ding

**Affiliations:** 1 Collaborative Innovation Center for Genetics and Development, State Key Laboratory of Genetic Engineering, Department of Microbiology, School of Life Sciences, Fudan University, Shanghai, China; 2 CAS Key Laboratory of Synthetic Biology, Institute of Plant Physiology and Ecology, Shanghai Institutes for Biological Sciences, Chinese Academy of Sciences, Shanghai, China; 3 Shanghai-MOST Key Laboratory of Disease and Health Genomics, Chinese National Human Genome Center at Shanghai, Shanghai, China

## Abstract

Growth of *Streptomyces* in submerged culture is characterized by the formation of complex mycelial particles, known as pellets or clumps, which strongly influence antibiotic production. Also, many bioactive molecules produced by *Streptomyces* have great potential to modulate soil bacteria morphological development. However, there has been no effort directed at engineering mycelial morphology using these small molecules. Here, thiostrepton was identified, using a combination of qRT-PCR, semi-preparative HPLC, and MALDI-TOF MS, as a pellet-inducing compound produced by *S. laurentii* ATCC31255. At sub-inhibitory concentration, thiostrepton stimulated *Streptomyces coelicolor* M145 pellet formation and antibiotics production were altered, with 3-fold and 2-fold decreases in actinorhodin and undecylprodigiosin yields, respectively. It was also shown that mycelial morphology can be influenced by other antibiotic class at sub-inhibitory concentrations. For instance, in the presence of spectinomycin, *S. coelicolor* M145, which under typical growth conditions forms large diameter pellets with many protruding hyphae, instead formed small diameter pellets with barely visible hyphae at the edge. Importantly, this morphology produced a 4-fold increase in undecylprodigiosin production and 3-fold decrease in actinorhodin production. These results indicated that these small molecules, previously identified as antimicrobials, also have great potential for influencing mycelial morphology.

## Introduction

Actinobacteria and, in particular *Streptomyces* species, show filamentous growth from single spores and are able to produce thousands of chemically complex, natural, bioactive metabolites. Most of the currently available antibiotics, as well as a large number of anticancer and antiparasitic agents, are produced by the genus *Streptomyces* ^1^. *Streptomyces* grow by the formation of a network of branching, multinucleate hyphae. In liquid culture, such as conditions used commercially to produce diverse bioactive metabolites, these branching hyphae become entangled and form large heterogeneous aggregates. Submerged growth of 145 reference species in liquid culture has revealed a continuum of morphological types, ranging from small fragmented particles to large pellets several millimeters in diameter^2^. During the fermentation process, these different mycelial morphologies lead to different degrees of nutrient and oxygen transfer within the pellets. Moreover, mycelial morphology directly influences the rheological properties of the fermentation broth, which in turn affects nutrient distribution within the reactor^3,4^. The linkage between mycelial morphology and antibiotics has been exemplified by *Streptomyces hygroscopicus* rapamycin production, where the formation of dispersed mycelial morphology is preferred^5^ and a high producing variant of *Streptomyces noursei* has been found to form dense pellets, while the wild type strain formed loose clumps^6^. In the literature, there is also a critical pellet size for erythromycin production^7^. Thus, it is of particular importance, through the application of genetic approaches, to engineer the mycelial morphology of industrial strains. To date, several genetic determinants involved in mycelial morphology have been identified in *Streptomyces*. These include the *cslA* gene that encodes a cellulose synthase-like protein that is required for pellet formation^8^, producing a much more dispersed growth by a *cslA* mutant than the wild type strain^9^, and *HyaS* that encodes a protein involved in cell-wall fusion. Deletion of *hyaS* has resulted in the formation of more loose pellets than those of the parental strain, which thus enhances undecylprodigiosin (Red) production^10^. Recently, van Dissel *et al*. have reported that the *mat* locus, which has been identified through reverse engineering, is key to mycelial morphology of *Streptomyces lividans*. Removal of the *matA* or *matB*, which encode polysaccharide synthases, yields a highly dispersed phenotype. The *matAB* null mutant of *S. lividans* produces more tyrosinase than the wild-type strain^11^. Genetic approaches based on understanding the biological processes that govern mycelial morphology and production might have potential for mycelial morphology regulation. However, classical genetic approaches are cumbersome and labor intensive and the generated morphology might not be preferred for product production. Therefore, a new methodology would be a useful alternative in practical applications.

In soil bacteria of many genera, multiple bioactive compounds are produced by a single species^12,13^. Many of these bioactive small molecules have been shown to regulate physiological and morphological development in soil bacteria^14–16^. The most prolific bioactive, small molecule producers are usually the actinobacteria and prime among these are the *Streptomyces*. A study by López has reported that purified nystatin produced by *Streptomyces noursei* induces biofilm formation in *B. subtilis* ^17^. In streptomycetes, the polyether antibiotic promomycin, as well as nigercin and monensin, have been shown to stimulate antibiotic production in *Streptomyces* spp.^18^. These observations raise the possibility that diverse, bioactive, small molecules secreted by *Streptomyces* might have some positive or negative influences on mycelial morphology.

The model actinomycete *S. coelicolor* A3(2) produces at least four chemically distinct classes of antibiotics, two of which are pigmented: blue actinorhodin (Act) and Red. In this study, a secondary metabolite secreted by *S. laurentii* ATCC31255, which lives in similar niches of *S. coelicolor* M145, was identified that stimulated pellet formation in *S. coelicolor* M145. Using quantitative real-time PCR (qRT-PCR), semi-preparative chromatography, and MALDI-TOF MS analysis, thiostrepton was identified as the compound produced by *S. laurentii* ATCC31255 that stimulated pellet formation of *S. coelicolor* M145. Furthermore, when purified thiostrepton at sub-inhibitory concentrations was added to *S. coelicolor* M145 cultures, facilitated pellet formation was accompanied by decreased production of Red and Act. Thereafter, the potentials of antibiotics from four other antibiotic classes were evaluated at subinhibitory concentrations. In the presence of chloramphenicol, erythromycin, and tetracycline, no differences were observed in mycelial morphology and antibiotic production, compared to the control group. When these bacteria were treated with spectinomycin, the formation of pellets with smaller diameter and barely visible hyphae at the surface were observed. Notably, this morphology accompanied a 4-fold increase in Red production but a 3-fold decrease in Act yield. The addition of various additives, ions and microparticles to the growth media has been reported to tailor bacterial morphology^4,19,20^. However, there has been no focus to date on engineering mycelial morphology using bioactive small molecules. Here, for the first time, regulation of mycelial morphology is reported through the addition of antibiotics.

## Results

### Regulation of *S. coelicolor* M145 morphology using genetic approaches

**Figure 1:**
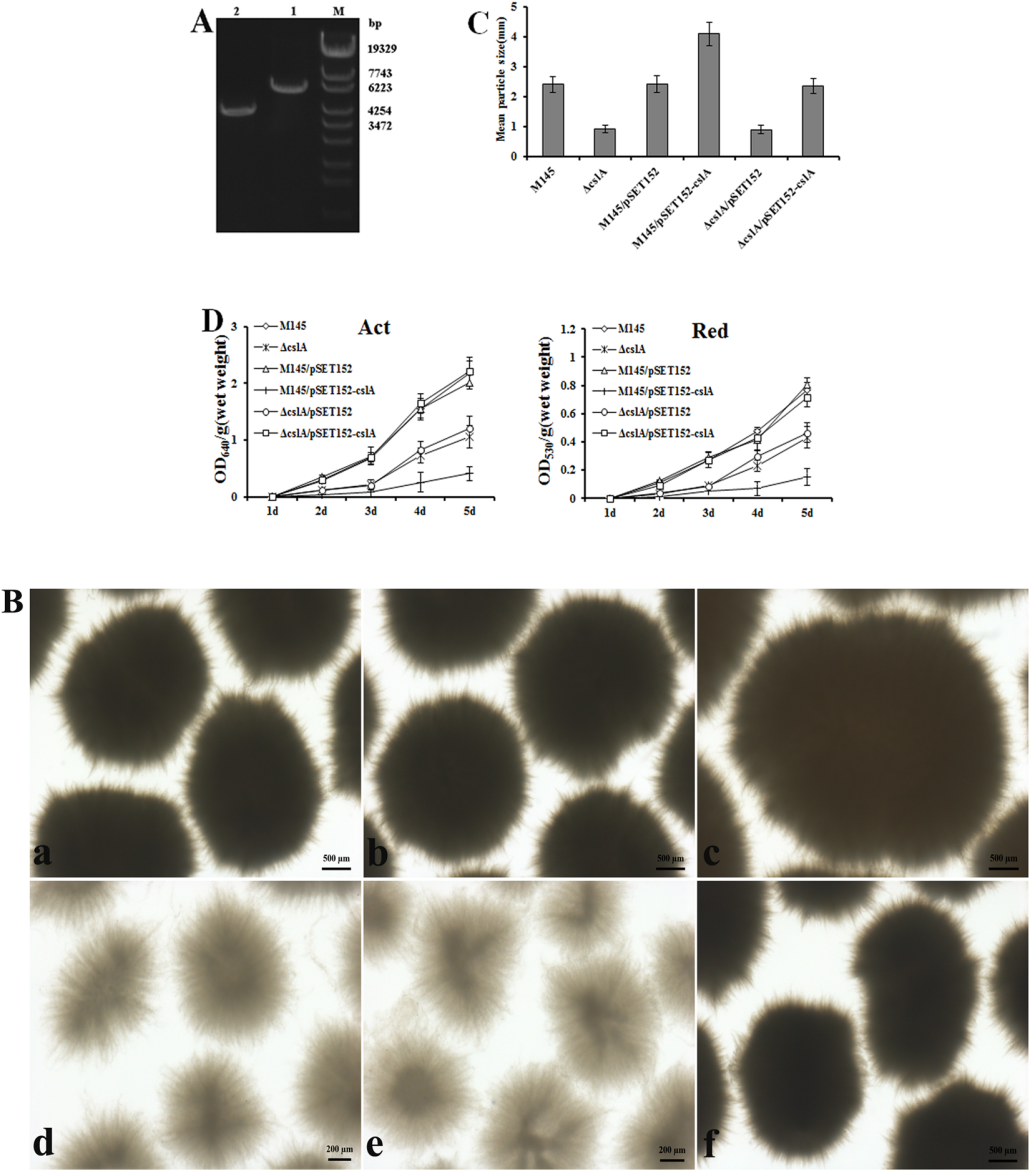
Morphology engineering by genetic approaches. (**A**) Confirmation of *S. coelicolor* M145 mutant for *cslA* by PCR using primers wh008-F and wh008-R. (M) marker, (1) 5897 bp amplified from wild type strain, (2) 4110 bp amplified from mutant strain. (**B**) Morphology of (a) wild type strain M145, (b) M145/pSET152, (c) M145/pSET152-cslA, (d) Δ*cslA*, (e) Δ*cslA/*pSET152, (f) Δ*cslA/*pSET152-cslA after five days of cultivation in TSB liquid medium. (**C**) Particles size of M145, M145/pSET152, M145/pSET152-cslA, Δ*cslA*, Δ*cslA/*pSET152, and Δ*cslA/*pSET152-cslA. (**D**) Act and Red production. Incubation was carried out in TSB liquid medium at 30 °C for five days. Data represented the average and standard deviation of three independent experiments.

As mentioned above, there exists a strong link between mycelial morphology and antibiotics production in streptomycetes, but the relationship between mycelial morphology and antibiotics production in *S. coelicolor* M145 was not clear. To ask whether dispersed mycelial morphology might be preferred by *S. coelicolor* M145 for Act and Red production, first a mutant for *cslA* gene was constructed and checked by PCR amplification (Fig. 1A). The wild strain and *cslA* mutant strain mycelial morphologies from shaken flask cultivation showed that the parental strain produced typical large diameter (2.45 ± 0.26 mm) particles with many protruding hyphae, whereas the *cslA* deletion strain resulted in the aggregation of particles with smaller diameters (0.90 ± 0.12 mm) and loose structure (Fig. 1B,C). Introduction of the *cslA* gene into the mutant strain completely abrogated the effects of the previous *cslA* deletion, confirming that *cslA* mutation underlay the observed phenotype (Fig. 1B,C). In antibiotic production, dispersed mycelial morphology produced ACT and Red at lower concentrations than the parental strain (Fig. 1D). We next investigated whether a different mycelial morphology favouring antibiotics production could be generated by overexpression of *cslA*. As shown in Fig. 1B,C, overexpression of *cslA* led to the observation of compact particles and an increase in particle diameter from 2.45 (±0.26) mm in parental strain to 4.11 (±0.35) mm. Notably, the presence of the empty vector pSET152 did not increase particle diameter in comparison to the wild-type strain. This mycelial morphology also failed to produce higher yields of Act and Red (Fig. 1D). Thus, these results indicated that dispersed mycelial morphology and compact particles with larger diameter were not suitable for Act and Red production.

### qPCR to identify fractions from *S. laurentii* culture supernatant

**Figure 2:**
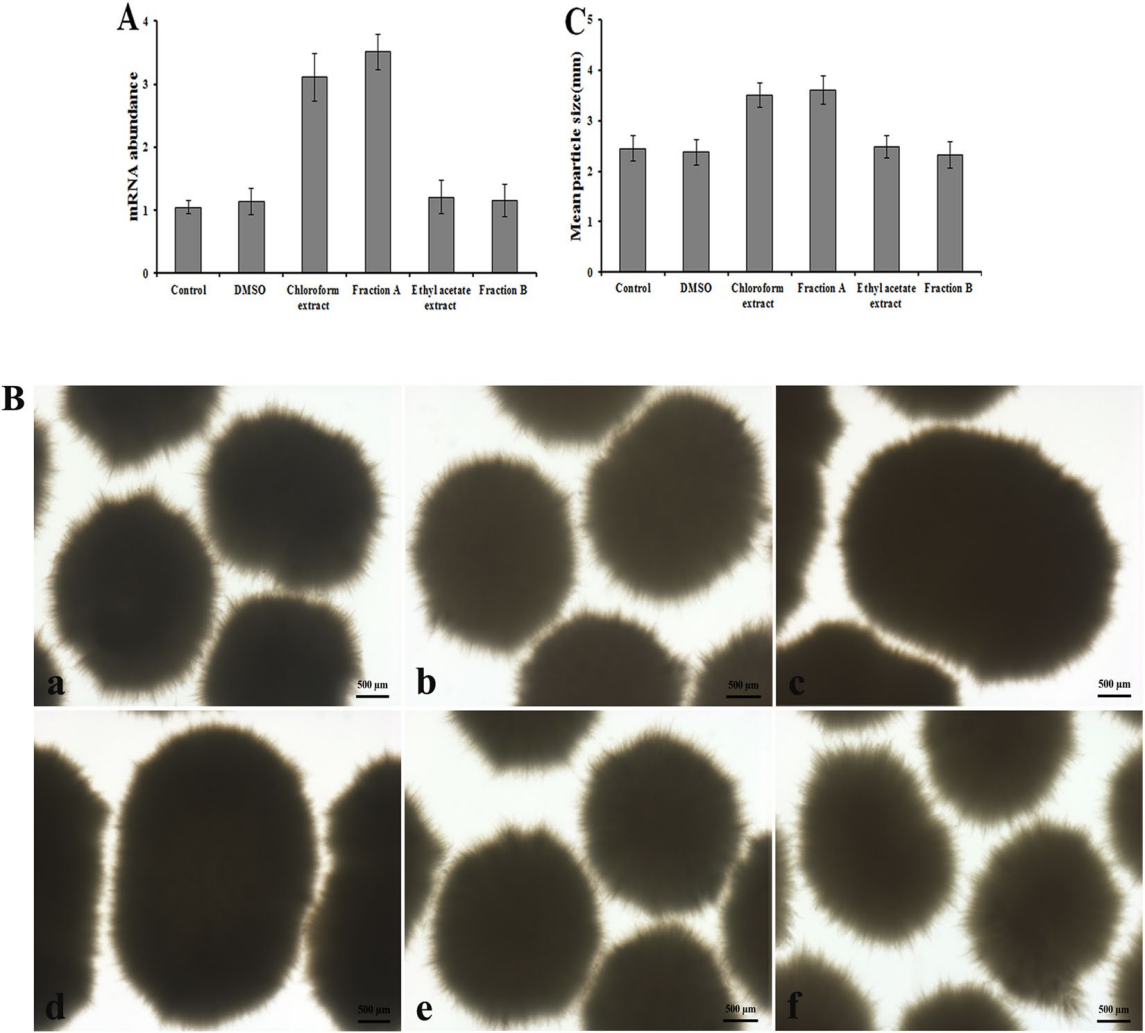
Mycelial morphology changes in response to the addition of bioactive molecules. (**A**) qRT-PCR detection of cslA transcripts on addition of the indicated amounts of DMSO, chloroform extract, fraction A, ethyl acetate extract, and fraction B (corresponding to 16th peak). Error bars represent the standard deviation of three independent experiments. (**B**) Influence of bioactive molecules supplementation on morphology of *S. coelicolor* M145 in submerged culture after inoculation with spores. (a) Control (b) DMSO, (c) Chloroform extract (d) Fraction A, (e) Ethyl acetate extract, (f) Fraction B. (**C**) Particles size for *S. coelicolor* M145 in submerged culture by addition of the bioactive molecules. Data represented the average and standard deviation of three independent experiments.

Soil bacteria produce a variety of diverse bioactive metabolites and many of them are instrumental in soil bacteria physiological and morphological development. However, whether these diverse, bioactive, small molecules are capable of regulating *Streptomyces* pellet formation and thus might be used for engineering mycelial morphology is unknown. To explore the possibility that these diverse metabolites produced by genus *Streptomyces* might play a role in pellet formation, *S. laurentii* ATCC31255, which lives in similar niches of *S. coelicolor* M145, was selected. Because of the high sensitivity and specificity of qRT-PCR, mycelial morphological responses of *S. coelicolor* M145 to the addition of culture supernatant extracts were first indirectly monitored by detecting the *cslA* expression level. The gene *hrdB*, which encodes the major vegetative sigma factor of *S. coelicolor* and expressed at relatively constant level throughout growth, was used as an internal control^21^. The mean *cslA* expression at the mRNA level was detected by qPCR in pellets (Fig. 2A), in which no transcriptional change in *cslA* was observed between an ethyl acetate extract treatment group and a control group after five days of cultivation. However, a chloroform extract treatment resulted in around 3-fold elevation in mRNA level of *cslA* (Fig. 2A). Mycelial particles resulting from culture extract treatments were also observed by stereomicroscope and analyzed by image analysis. Compared to the control group, treatment with the chloroform extract resulted in the formation of larger and denser pellets (Fig. 2B,C). The group treated with the ethyl acetate extract showed no change in mycelial morphology, compared to the control group. The chloroform extract, with an observed potent stimulatory activity for *S. coelicolor* M145 pellet formation, was selected for further purification.

### Isolation of bioactive molecules by semipreparative HPLC

**Figure 3:**
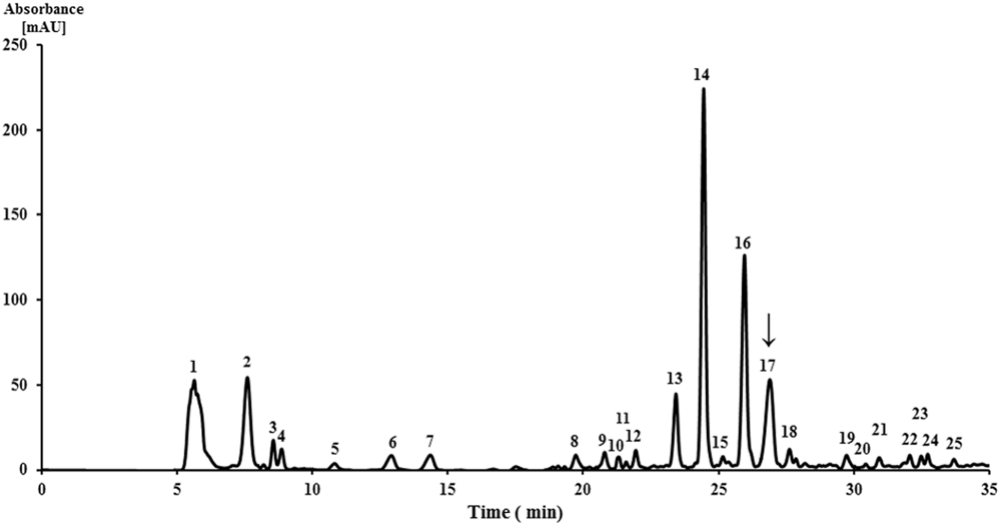
Elution profile of chloroform extract on semipreparative HPLC column. UV detected at 254 nm. The arrow indicates the fraction A, which corresponds to the 17th peak.

Potent stimulatory molecules produced by *S. laurentii* ATCC 31255 were isolated using semipreparative HPLC purification. The elution profile of the chloroform crude extract yielded 25 fractions (Fig. 3) and the stimulation activity of each fraction against *S. coelicolor* M145 pellet formation was evaluated using qRT-PCR as the screening method. A solution of fraction A at 1.5 mg/mL, corresponding to the 17th peak (retention time 26.5 min) in Fig. 3, was found to be the only potent stimulatory agent from the crude extract (Fig. 2). The other fractions did not show any significant stimulatory activity at the applied concentrations (results not show).

### MALDI-TOF analysis and minimal inhibitory concentration determination

**Figure 4:**
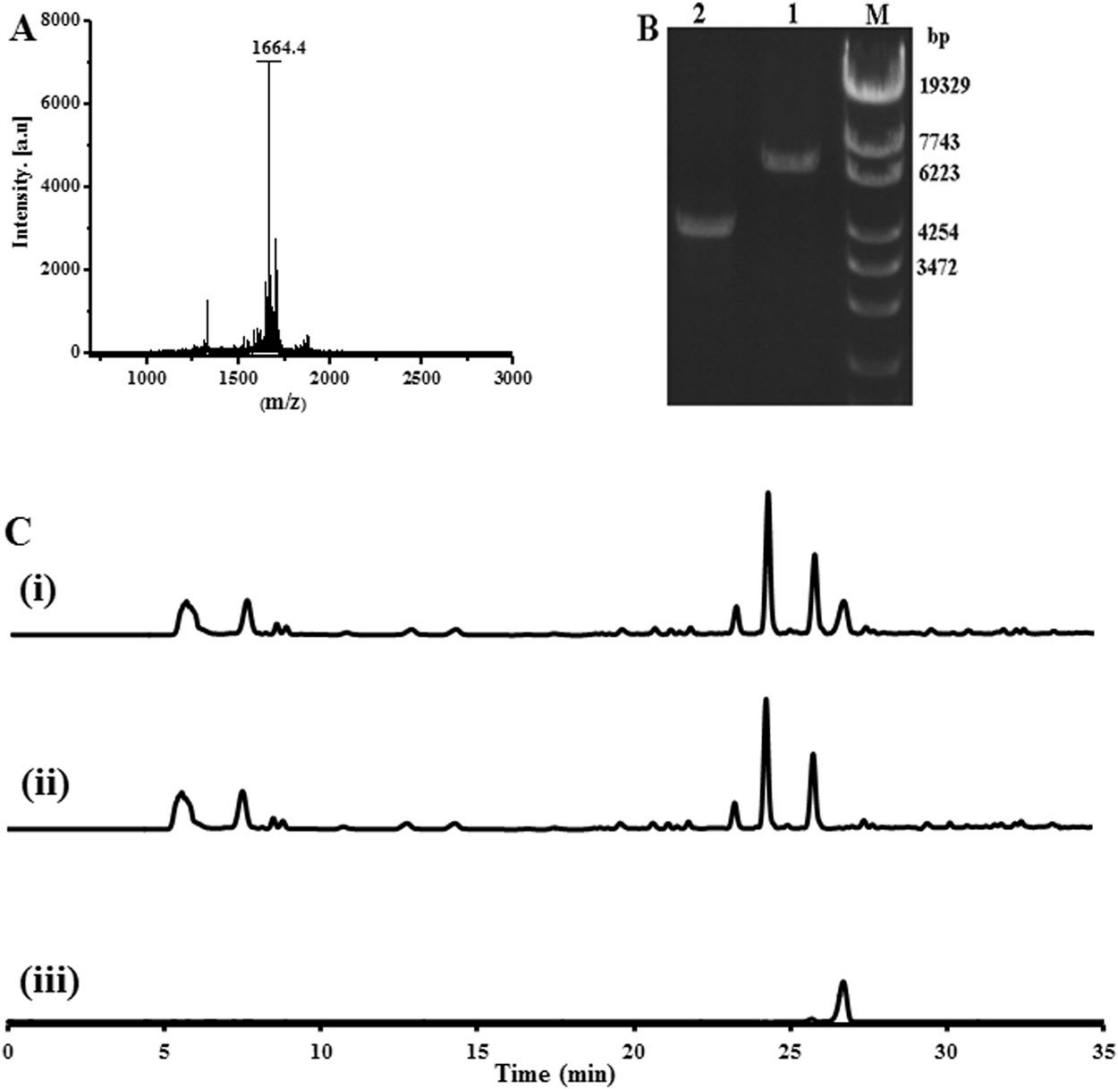
Identification of the bioactive small molecule. (**A**) MALDI-MS spectra of the fraction A. (**B**) PCR confirmation of *S. laurentii* mutant for *tsrJ* by using primers wh015-F and wh015-R. (M) marker, (1) 6717 bp amplified from wild strain of *S. laurentii*, (2) 4061 bp amplified from mutant strain. (**C**) HPLC analysis of the extracts from the culture of (i) wild type strain M145 (ii) mutant strain (iii) thiostrepton. Absorbance was monitored at 254 nm.

For molecular weight determination by MALDI-TOF, the purity of the collected fraction A was confirmed by reinjection into HPLC. The mass ion, m/z 1664.4, was observed in fraction A (Fig. 4A) and compared with available reference compounds and published literature. A search of known compounds produced by *S. laurentii* with this molecular weight yielded thiostrepton as a potential match. Furthermore, the mutant strain resulting from an in-frame deletion of gene *tsrJ*, which encodes a dehydratase required for thiostrepton biosynthesis (Fig. 4B), completely lost its ability to produce fraction A (Fig. 4C). Thiostrepton purchased from Sigma-Aldrich, Inc. (St. Louis, MO, USA) produced a peak with the same retention time of 26.5 min (Fig. 4C). These results indicated that the ions observed in the MALDI-TOF MS data represent thiostrepton. This result was surprising because thiostrepton is well known to have antimicrobial activity. Next, the minimal inhibitory concentration (MIC) of thiostrepton was determined by inoculating spore solutions into TSB medium containing various thiostrepton concentrations. The MIC concentration (1200 nM) was significantly higher than the concentration at which increased level of *cslA* transcript was found (30 nM).

### Purified thiostrepton stimulated pellet formation

**Figure 5:**
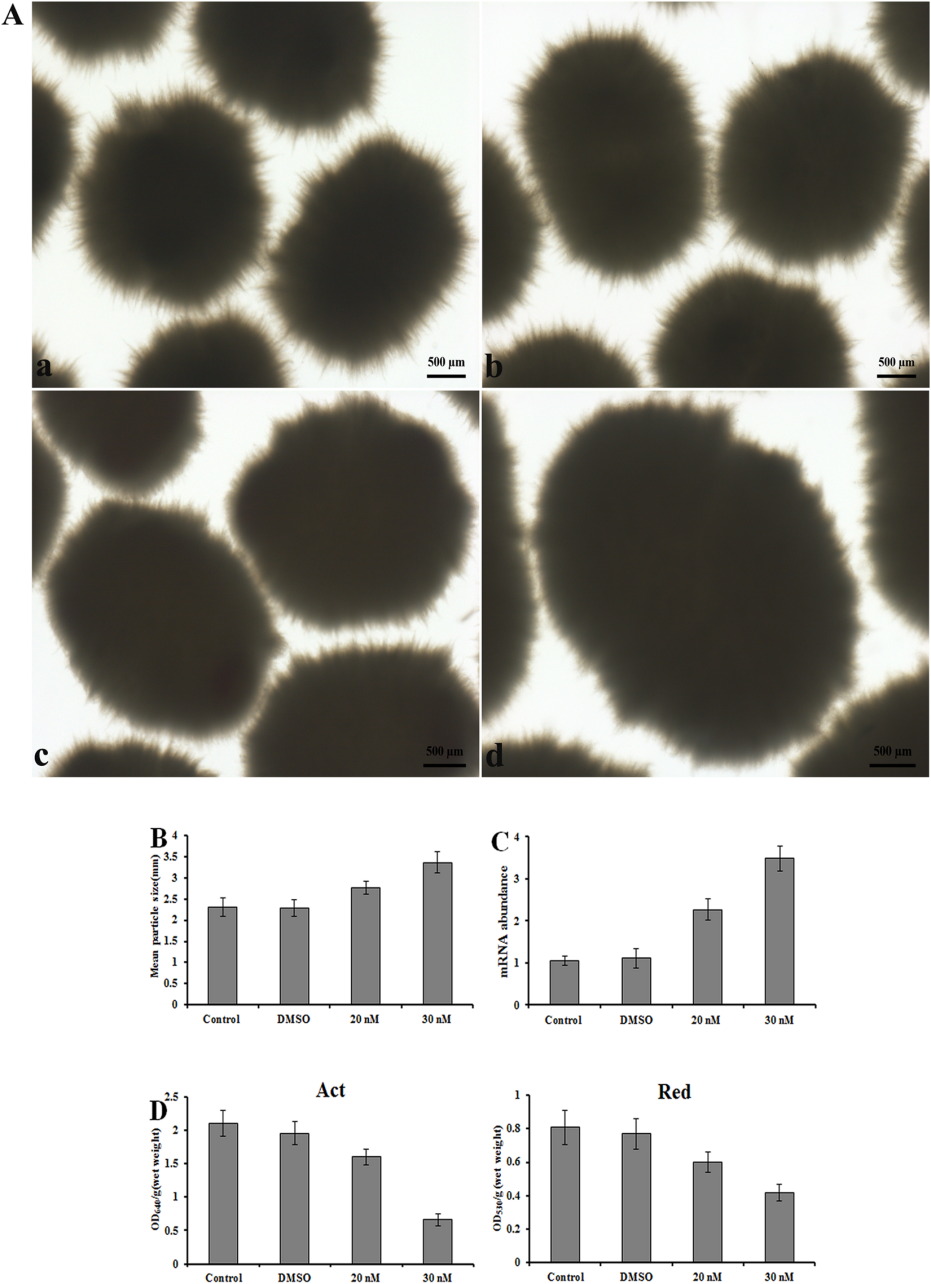
Purified thiostrepton stimulated the *S. coelicolor* M145 pellet formation. (**A**) Morphology of *S. coelicolor* M145 after five days of cultivation in the presence of thiostrepton. (a) control, (b) DMSO, (c) 20 nM, (d) 30 nM. (**B**) Particles size of *S. coelicolor* M145 in the presence of thiostrepton. (**C**) The effect of different concentrations of thiostrepton on cslA mRNA transcripts in *S. coelicolor* M145 pellets. (**D**) Act and Red production in the presence of thiostrepton. Data represented the average and standard deviation of three independent experiments.

To further confirm these findings, purified thiostrepton at a final concentration of 30 nM, or DMSO as a control, was added to the culture medium and the effect on pellet formation assessed. Samples were taken from five days cultures in TSB and observed using a stereomicroscope. Representative images for microscopy from strains with and without thiostrepton showed that, compared to the control group, treatment by thiostrepton led to the formation of pellets with larger diameter and compact surfaces (Fig. 5A,B), which was in agreement with qRT-PCR results (Fig. 5C). Notably, this mycelial morphology also showed significantly less Act and Red production, with 3-fold and 2-fold decreased yield, respectively (Fig. 5D). Likewise, when 20 nM of thiostrepton was added to the culture medium, a decrease in the mean pellet diameter coupled with an increase in antibiotics production was observed, but to a less extent than at 30 nM, thereby demonstrating the dose-dependence of the thiostrepton effect.

### Morphology engineering by different classes of antibiotics

**Figure 6:**
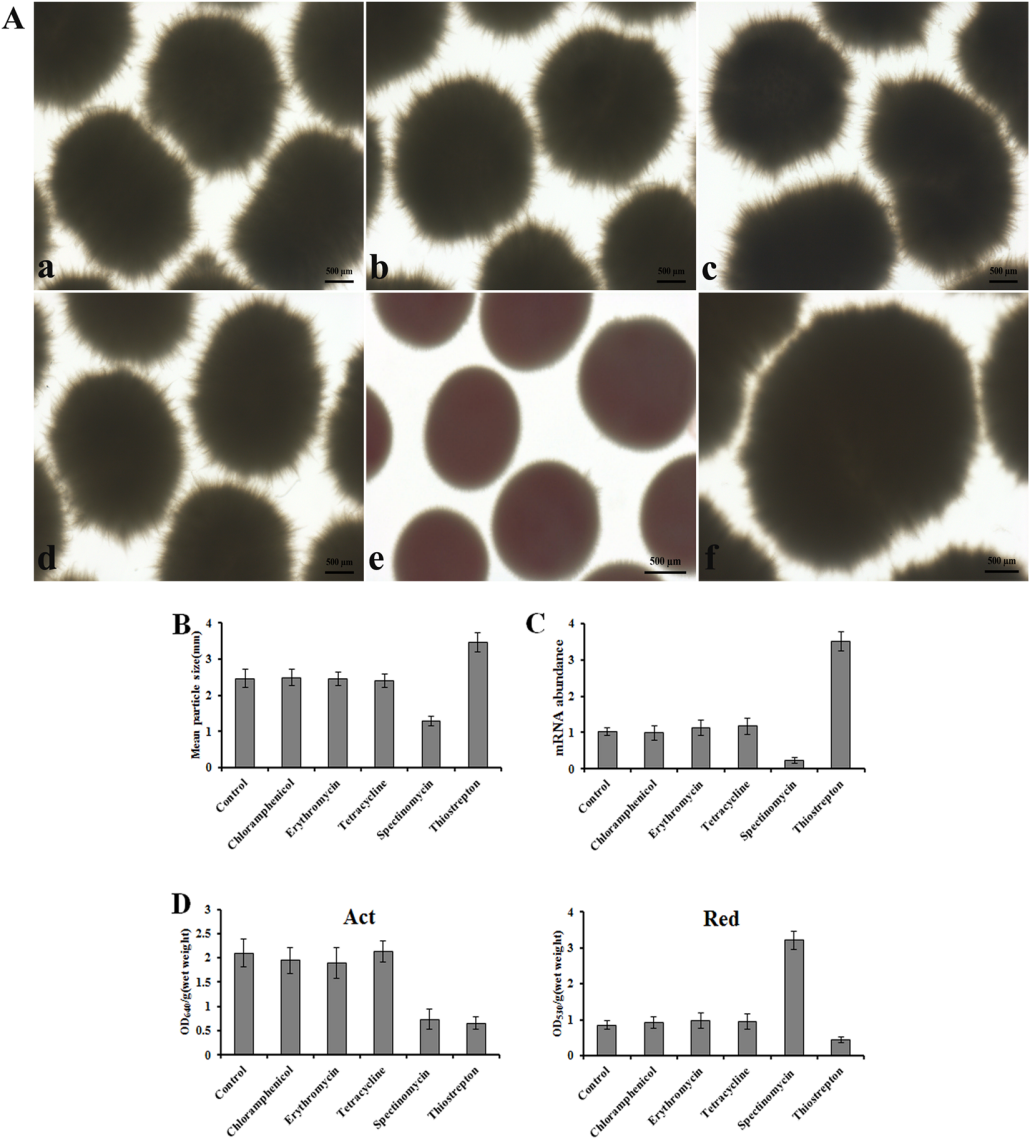
Morphology enginnering by different classes of antibiotics. (**A**) Morphology of *S. coelicolor* M145 by treatment with antibiotics. (a) control, (b) chloramphenicol, (c) erythromycin, (d) tetracycline, (e) spectinomycin, (f) thiostrepton. (**B**) Particles size for *S. coelicolor* M145 at sub-inhibitory concentrations of antibiotics. (**C**) qRT-PCR detection of cslA transcripts on addition of different classes of antibiotics. (**D**) Act and Red production by *S. coelicolor* M145 in the presence of different classes of antibiotics. Data represented the average and standard deviation of three independent experiments.

Having found that thiostrepton at sub-inhibitory concentrations stimulated pellet formation, we next tested whether antibiotics from other different classes could function and four commercially available antibiotics chloramphenicol, tetracycline, spectinomycin, and erythromycin were evaluated. The results of data analysis showed that these antibiotics exhibited an MIC of 190, 20, 200, and 10 µM respectively. For treatment with these antibiotics, 1/40^th^ of each MIC was also used. After five days of cultivation in the presence of chloramphenicol, tetracycline, spectinomycin, erythromycin, or thiostrepton, the corresponding pellets were transferred to Petri dishes and observed by stereomicroscope. Mycelial morphology was apparently unchanged in groups with added chloramphenicol, tetracycline, and erythromycin, compared with that of wild-type strain M145 (Fig. 6A,B). For Red and Act production, no difference in strains with or without antibiotic treatment was observed (Fig. 6D). However, in the spectinomycin treatment group, the typical large diameter particles with many protruding hyphae in the control group, were replaced with smaller diameter pellets with barely visible hyphae at the edge (Fig. 6A,B), and the corresponding mRNA level of cslA decreased dramatically (Fig. 6C). Interestingly, Red production increased 4-fold, compared to the control, whereas an apparent decrease in Act production concurrent with mycelial morphology alteration was noticed (Fig. 6D).

## Discussion

Thiostrepton is a natural cyclic oligopeptide antibiotic of the thiopeptide class, which has long been known to be a potent bacteriocide of Gram-positive bacteria^22,23^. The present work demonstrated that thiostrepton at sub-inhibitory concentrations stimulated pellet formation in *Streptomyces coelicolor* M145. Thiostrepton at these concentrations was shown able to induce pellet gene expression and thus form denser pellets. The view that sub-MIC antibiotic concentrations act as signals in bacterial morphological development is not new and has been discussed^24^. Nevertheless, here, treatment with thiostrepton at 1/40^th^ of MIC led to the formation of larger pellets with compact surfaces. Also, when a lower thiostrepton concentration (1/60^th^ MIC) was added to the culture medium, a decrease in mean pellet diameter was observed, but to a less extent than at 1/40th MIC, thereby demonstrating the dose-dependence of thiostrepton effect. More was learned about the function of antibiotics in *Streptomyces* pellet formation by adding four other antibiotics from different antibiotic classes to the culture media at the time of inoculation. The results indicated that bacteria formed compact pellets with smaller diameter in the presence of spectinomycin which suggested that this aminoglycoside antibiotic at sub-inhibitory concentrations could be used for mycelial morphology modifications. However, it should be noted that aminoglycoside antibiotic exposure leads to high levels of spontaneous mutants^25,26^. In *E. coli*, spectinomycin has been reported to corrupt ribosomes and cause protein misfolding^27^. Intriguingly, treatment with spectinomycin elicited a dramatic decrease in cslA mRNA abundance relative to the control. This data implied that spectinomycin affected the gene expression involved in mycelial morphology formation at the transcriptional level in addition to a possible effect at the translational level. It was also noteworthy that only spectinomycin and thiostrepton have been shown to influence *cslA* expression among the five different antibiotic classes tested. Why exposure of thiostrepton and spectinomycin might affect *cslA* expression is an interesting question that should be addressed in future studies. Future efforts will be directed towards deciphering the molecular basis of mycelial morphological changes in response to the addition of sub-inhibitory antibiotic concentrations in submerged culture.

The mycelial life style of *Streptomyces*, resulting in the formation of unfavorable pellets, has been a major bottleneck in their commercialization and, over decades, efforts have been made to select strains with better growth properties for production. Dispersed mycelial morphology is generally favorable in terms of growth rate and biomass accumulation. For rapamycin production, the formation of dispersed mycelial morphology in liquid culture helps productivity in the bioreactor^5^ while for the production of some antibiotics pelleted growth is preferred. For instance, the formation of compact pellets has been observed to be a prerequisite for hybrid antibiotic production^28^, and erythromycin production by *Saccharopolyspora erythraea* requires larger pellets for optimal yields^7^. In the present study, dispersed mycelial morphology was initially proposed to be preferred by *S. coelicolor* M145 for Act and Red production. However, *cslA* gene deletion or overexpression, which was associated with dispersed or large pellet morphology, respectively, led to reduced Act and Red production. Interestingly, pellet formation with smaller diameter and barely visible hyphae at the surface led to increased Red production but a decline in Act production. Hence, optimal morphology varied from product to product. The optimal kind of mycelial morphology for specific antibiotic formation remains an intriguing question that should be addressed in future studies. The classical biochemical process parameters, such as agitation rate, temperature, medium composition, pH, and inoculum concentration, have great influence on mycelial morphology^29^. Perhaps antibiotic treatments in combination with these various parameters could help us to make the evaluation.

Present studies largely apply the molecular genetic approaches to modify mycelial morphology of industrial strains. However, this and previous studies have indicated that genetic operations are inconvenient and might later slow down further improvement. Moreover, to date, the genes involved in mycelial morphology are poorly understood in *Streptomyces* submerged cultures^8,10,11^. In contrast, soil bacteria have evolved and maintained the ability to produce diverse, bioactive, small molecules in natural environments, indicating that such an activity is useful for regulation of their social behavior and maintenance of multicellular communities^30^. The diversity of *Streptomyces* and related bacteria metabolites might play a key role in fine-tuning the timing and strength of gene expression involved in physiological and morphological development. In *B. subtilis*, siderophores produced by multiple fungi and streptomycetes have been shown to promote sporulation^31^. In *S. coelicolor*, a hydrophobic peptide SapT, produced by the soil microbe *Streptomyces tendae*, has been shown to stimulate aerial hyphae formation^32^. However, to the best of our knowledge, these bioactive, small molecules have not been examined for the possible regulation of mycelial morphology. Importantly, in this study, the treatment of thiostrepton and spectinomycin at sub-inhibitory concentrations resulted in considerable alteration of mycelial morphology in *S. coelicolor* M145. Therefore, these findings opened new avenues for modifying *Streptomyces* mycelial morphology and future studies should focus on the potential of a variety of secondary metabolites produced by *Streptomyces* and related bacteria for mycelial morphology engineering.

In summary, we reported for the first time that modification of mycelial morphology by sub-inhibitory concentrations of antibiotics. The presence of thiostrepton resulted in the observation of pellets with larger diameter and compact surface, whereas treatment with spectinomycin led to the formation of the pellets with smaller diameter and barely visible hyphae at the edge. Our results indicated these diverse metabolites initially identified as antimicrobial can also act as versatile tools for engineering mycelial morphology in *Streptomyces*.

## Methods

### Strains, plasmids, and culture conditions

Plasmid pKC1139 was a kind gift from Professor Hong Tan (Chengdu Institute of Biology, Chinese Academy of Science). *Streptomyces laurentii* ATCC31255 was kindly provided by Professor Wen Liu, the Shanghai Institute of Organic Chemistry, Chinese Academy of Sciences, China. *Streptomyces coelicolor* A3 (2) M145 was cultured and stored in our lab. *S. coelicolor* M145 and *S. laurentii* ATCC31255 were grown at 30 °C on mannitol-soya flour (MS) agar plates or in liquid TSB medium^33^. Stock solutions of chloramphenicol, tetracycline, spectinomycin, erythromycin, and thiostrepton purchased from Sigma-Aldrich were made according to the manufacturers’ instructions.

### Generation of mutant strains

The homologous arms of *cslA* gene were separately amplified from genomic DNA of *S. coelicolor* M145 with the primer pairs, *cslA* up arms-F and *cslA* up arms-R, and *cslA* down arms-F and *cslA* down arms-R. After digesting pKC1139 with Hind III, the two amplicons were fused into the Hind III site of the pKC1139 vector using the ClonExpress MultiS One Step Cloning Kit (Vazyme, Nanjing, China) to generate the plasmid pWH1005. Similarly, the homologous arms of *tsrJ* gene were separately amplified from the genomic DNA of *S. laurentii* ATCC31255 with the primer pairs, wh012-F and wh012-R, and wh013-F and wh013-R. After digestion of pKC1139 with Hind III, the two arms were fused into the Hind III site of pKC1139 vector to generate the plasmid pWH1006.

All primers used for plasmid construction are shown in Table 1. Conjugation of *E. coli* ET12567/pUZ8002 with *Streptomyces* was performed as described previously^33^. For the deletion of *tsrJ* and *cslA*, colonies that were apramycin resistant at 37 °C were identified as the integrated mutants. Apramycin resistant single crossover integrated mutants were subcultured by three rounds of streaking in the absence of apramycin at 37 °C to obtain double-crossover recombinants. Colonies with apramycin sensitive phenotypes were selected and the deletion of *cslA* and *tsrJ* was checked by PCR and DNA sequencing, leading to the identification of Δ*cslA* and Δ*tsrJ* recombinant strains.

**Table 1:** Primers used for qPCR analysis of mRNA and genetic engineering.

Primer	Primer Sequence(5′-3′)
hrdB-F	tctctgtcatggcgctcattg
hrdB-R	ttccactgagtggccggaatc
SCO2836-F	gccaagtggaaccggaagaag
SCO2836-R	acggaggcgaagaagtcgtag
cslA-up-arms-F	cgacggccagtgccaagctttctccgggttcgttgacgatg
clsA-up-arms-R	accaagggcgatctcatatgtcgtacttcgggagcgtcttc
cslA-down-arm-F	catatgagatcgcccttggtggacttg
cslA-down-arms-R	tcgacctgcagcccaagcttgactgcaccaggttcttaccc
wh008-F	tggtggcctcgttgtggatg
wh008-R	acgcctacctctgggtcaag
wh009-F	tagagtcgacctgcagcccaag
wh009-R	gaattcgtaatcatgtcatagc
wh010-F	gaccggtgcaggcgaaggaag
wh010-R	tatgacatgattacgaattctcattccttacgtcccccaag
wh011-F	tgggctgcaggtcgactctagatcgcaggtgcacgcggtcg
wh011-R	cttccttcgcctgcaccggtcggatcctaccaaccggcacg
wh012-F	cgacggccagtgccaagcttgagccgagtcgttcggaattg
wh012-R	tcctcggtgaaccgagatctgaggacaactgggcgatgatg
wh013-F	agatctcggttcaccgaggaattcatc
wh013-R	tcgacctgcagcccaagcttgccctggtagtacgtgttgtc
wh015-F	gctctccactgtcacggaaac
wh015-R	caggtagaagagcgtgtaccg

### Overexpression and complementation of *cslA*

The vector pWH1007, a plasmid allowing *cslA* overexpression, was constructed as follows. The pSET152 vector was first linearized by PCR amplification using primer wh009-F and wh009-R. The gene *cslA* and promoter ermEp^*^ were then amplified from genomic DNA of *Streptomyces coelicolor* M145 and *Saccharopolyspora erythraea* NRRL2338, respectively. Briefly, *cslA* was amplified using primers wh010-F and wh010-R. Promoter ermEp^*^ was amplified using primers wh011-F and wh011-R. The above three PCR products were mixed and ligated using ClonExpress MultiS One Step Cloning Kit to generate pWH1007, with *cslA* placed downstream of ermEp^*^. The plasmid pWH1007, along with the empty vector pSET152 were then conjugated from the donor *E. coli* ET12567/pUZ8002 into the parental strain M145 and Δ*cslA* strain. As a result, recombinant strains of M145/pSET152, M145/pSET152-cslA, Δ*cslA/*pSET152, and Δ*cslA/*pSET152-cslA were obtained. The primers used for constructing the plasmids are shown in Table 1.

### Preparations of culture extracts

For the production of bioactive, small molecules from *S. laurentii*, 30 mL of TSB was inoculated into 250 mL Erlenmeyer flasks with 2 x 10^6^ spores/mL and incubated at 30 °C and 200 rpm for 48 h. Then, 5 mL of the seed culture was inoculated into two 2 L flasks containing 500 mL TSB and incubated in a rotary incubator at 30 °C and 200 rpm for five days. The culture medium was centrifuged at 8000 g and 4 °C for 15 min and the supernatant collected. One of the two culture supernatants was extracted with an equal volume of chloroform and the other with an equal volume of ethyl acetate. The chloroform and ethyl acetate layers were concentrated *in vacuo* to produce dried extracts. The dried products were dissolved in 1 mL of 100% dimethyl sulfoxide (DMSO) and filtered through sterile filter membranes (0.45 µm). The solutions were then used for further experiments.

### qRT-PCR detection of gene expression in pellets

For RNA extraction from *S. coelicolor* M145, 20 µL of the solubilized extracts were added to 30 mL of TSB medium and inoculated into 250 mL Erlenmeyer flasks with 1 x 10^6^ spores/mL and incubated at 30 °C, and 200 rpm for five days. Flasks with the addition of an equal volume of sterilized DMSO were prepared as controls, as described above. Pellets grown in flasks at 30 °C for five days were collected, and a modified Kirby mixture was used for RNA isolation^33^. The isolated RNA was then subjected to phenol/chloroform extraction and RNase-free DNase I treatment, as described previously^33^. One microgram of the resulting RNA was used to generate cDNA, using the Quant Script RT kit (Tiangen Biotechnology Co., Ltd., Beijing, China). The mRNA levels of *cslA* and *hrdB* were then detected using an SYBR green-based quantitative RT-PCR. The primer pairs for *cslA* were SCO2836-F and SCO2836-R and for *hrdB* were hrdB-F and hrdB-R. The primers used for qRT-PCR are shown in Table 1. The program for amplification was as follows: a denaturation step at 95 °C for 5 min, followed by 40 cycles of 95 °C for 15 sec, and 60 °C for 30 sec for annealing and extension. For determination of mRNA levels of the target gene relative to the *hrdB* control, the relative quantitative 2^−ΔΔCt^ method was used^34^.

### Isolation of bioactive small molecules by semipreparative HPLC

For bioactive molecule isolation, 10 L of culture broth was centrifuged for 15 min at 8000 g and 4 °C for 15 min. The collected supernatant was then extracted with an equal volume of chloroform, concentrated under vacuum, and redissolved in DMSO for further isolation. HPLC purification was carried out on an Ultimate XB-C18 column (10 × 250 mm, 5 µm particle size, Welch, Shanghai, China), and developed with the followed program: 0 to 10 min, constant 80% solvent A (H_2_O containing 0.1% trifluoroacetic acid, TFA) and 20% solvent B (acetonitrile, ACN); 10 to 35 min, a linear gradient from 80% solvent A and 20% solvent B to 10% solvent A and 90% solvent B. Detection was at 254 nm and the flow rate at 2.4 mL/min using an Aligent 1200 HPLC system (Agilent Technologies, Inc.). Fifty microliters of crude extract were loaded onto the C_18_ RP-HPLC column and, according to the elution profile, 25 fractions were collected manually. The HPLC eluent fractions were combined and collected into vials. The fractions were then dried under reduced pressure at 30 °C and purity of the isolated fractions confirmed by reinjection into the HPLC. For further screening of the bioactive molecules by qRT-PCR, each fraction was redissolved in 1 mL of DMSO. The solutions were then diluted 20-fold in DMSO, and 20 µL of each and 30 mL TSB medium inoculated into 250 mL Erlenmeyer flasks and incubated at 30 °C and 200 rpm for five days.

### MALDI-TOF-MS analysis

MALDI-TOF-MS measurements were performed on a Bruker Ultraflex TOF/TOF MS (Bruker, Bremen, Germany) using the Bruker Flex Analysis Software (version 2.4). The fraction A sample solution (1 µL) was spotted onto the MALDI target plate. 1 µL of CHCA matrix solution at a concentration of 10 mg/mL in 70% ACN with 0.1% TFA was then overlaid and dried in air. All spectra were obtained in positive reflector mode.

### Determination of minimum inhibitory concentrations (MICs) and quantification of antibiotics

All MIC assays were performed in TSB supplemented with various concentrations of each antibiotic. Growth was assessed after incubation at 30 °C for 48 h. The MIC was defined as the lowest concentration of antibiotic at which no visible growth was observed. Act and Red Production in liquid medium was determined as described by Kieser *et al*.^33^. Briefly, 30 mL of grown culture of each group was filtered to separate the pellet and supernatant. To determine the Act, the supernatant was treated with KOH (1 M final concentration), and A_640_ of the supernatant was determined. To determine the Red, the pellet was dried under a vacuum and extracted with methanol overnight at room temperature. The methanol was acidified with HCl (0.5 M final concentration). The Red production was determined by measuring A_530_.

### Image analysis by microscopy

After five days of cultivation, the mycelial morphology was analyzed using a Zeiss Axio Zoom.V16 stereomicroscope with an AxioCam MRc camera. The particles of each group were transferred to a Petri dish containing diluted medium. 500 particles were analyzed for each group. For each group, several images of particles were obtained. The ImageJ software was used for Image analysis.

### Data availability statement

All data generated or analysed during this study are included in this published article.
